# Clinical and radiological outcomes of a modified anatomic posterolateral corner reconstruction technique using a single semitendinosus autograft

**DOI:** 10.1007/s00402-023-04862-6

**Published:** 2023-05-04

**Authors:** Ahmed Helal, Abdelhakim E. Marei, Ahmed Shafik, Elsayed Elforse

**Affiliations:** Department of Orthopaedics, Tanta Faculty of Medicine, Tanta University, El-Gash St. Medical Campus, Tanta, El-Gharbia Governorate Egypt

**Keywords:** Posterolateral corner injury, Knee dislocation, Multiligament knee injury, Posterior cruciate ligament, Anterior cruciate ligament

## Abstract

**Purpose:**

We aimed to assess the clinical and radiological outcomes of a modified anatomical posterolateral corner (PLC) reconstruction technique using a single autograft.

**Methods:**

This prospective case series included 19 patients with a posterolateral corner injury. The posterolateral corner was reconstructed using a modified anatomical technique that utilized adjustable suspensory fixation on the tibial side. Patients were evaluated subjectively using the international knee documentation form (IKDC), Lysholm, and Tegner activity scales and objectively by measuring the tibial external rotation angle, knee hyperextension, and lateral joint line opening on stress varus radiographs before and after surgery. The patients were followed-up for a minimum of 2 years.

**Results:**

Both IKDC and Lysholm knee scores significantly improved from 49 and 53 preoperatively to 77 and 81 postoperatively, respectively. The tibial external rotation angle and knee hyperextension showed significant reduction to normal values at the final follow-up. However, the lateral joint line opening measured on the varus stress radiograph remained larger than the contralateral normal knee.

**Conclusion:**

Posterolateral corner reconstruction with a hamstring autograft using a modified anatomical reconstruction technique significantly improved both the subjective patient scores and objective knee stability. However, the varus stability was not completely restored compared with the uninjured knee.

**Level of evidence:**

Prospective case series (Level of evidence IV).

## Introduction

Posterolateral corner (PLC) injuries account for approximately 10% of acute ligamentous knee injuries [1] and often occur in combination with anterior cruciate ligament (ACL) or posterior cruciate ligament (PCL) tears; isolated injuries represent only 28% of all PLC injuries [2].

PLC injuries frequently occur due to a hyperextension force combined with varus and/or external rotation of the tibia [3]. The most important components of the PLC are the lateral collateral ligament (LCL), popliteofibular ligament (PFL), and popliteus tendon (PT). The LCL acts as the primary static stabilizer against varus angulation at all flexion angles. The PFL and PT resist external tibial rotation, with the PT providing additional secondary stabilization against posterior tibial translation and internal rotation [4, 5].

Untreated PLC injuries have detrimental long-term effects and may lead to chronic pain, instability, varus thrust, and even failure of the reconstructed ACL and PCL grafts [6].

Numerous techniques have been described to repair, augment, and reconstruct the PLC [7–11], including primary repair, biceps tenodesis, biceps augmentation, and fibular- and tibial-based reconstructions [12]. A growing consensus in the literature favors reconstruction over repair [13, 14].

One of the most common techniques for PLC reconstruction is the fibular sling procedure with a single femoral tunnel using a single graft, which is often referred to as the Larson procedure [15].

Laprade et al. [16] introduced the term anatomical reconstruction, in which the three main components of the PLC were recreated (LCL, PFL, and PT) using a split-thickness Achilles tendon allograft. The primary advantage of this technique is the reconstruction of a popliteal bypass graft, which has superior biomechanical properties to that of fibular-based reconstructions [17].

**Fig. 1:**
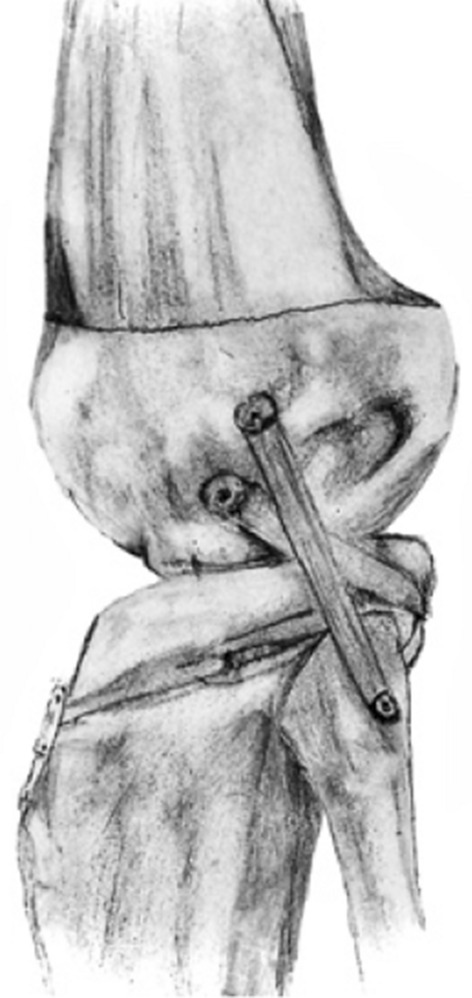
Schematic drawing of the modified anatomical posterolateral corner reconstruction technique

The main disadvantages of Laprade’s anatomical reconstruction are the need for two grafts and the unavailability of allografts in several countries. To overcome these hurdles, we utilized a modified anatomical technique using a single hamstring autograft with adjustable loop fixation on the tibial side to reconstruct the PLC (Fig. 1). The main goal of our study was to investigate the subjective and objective results of this technique. We hypothesized that this technique would restore knee stability and improve patient-reported outcomes.

## Material and methods

This case series was prospectively designed for consecutive patient recruitment. This study was approved by the Ethics Committee of faculty of medicine, Tanta university, Egypt, and conducted according to the principles of the Declaration of Helsinki. All the patients provided written informed consent to participate in the study. Between May 2018 and April 2020, 19 consecutive patients underwent PLC reconstruction using a modified anatomical posterolateral reconstruction technique.

All the following criteria have to be met to be included in the study: posttraumatic varus instability with pathological lateral joint line opening at 20° of knee flexion, > 4 mm of varus stress-induced gapping on radiographs at 20° of knee flexion in comparison to the uninjured knee, positive dial test at 30° of knee flexion (> 10° side-to-side difference [SSD] of external tibial rotation) suggesting a posterolateral rotatory instability in a patient presenting with functional instability or pain. The included patients had to have a normal valgus or primary varus alignment of the acutely injured knee. The exclusion criteria were advanced knee arthritis (grades 3–4 Kellgren–Lawrence classification), revision surgeries, common peroneal nerve injury, injured or previously operated contralateral knee, chronic cases with uncorrected primary varus malalignment (mechanical axis lies medial to the medial tibial spine with a hip–knee–ankle angle < − 3), and double or triple varus knees with a varus thrust gait that was not corrected by an osteotomy procedure.

### Evaluation and rating scales

All patients involved in the study completed the International Knee Documentation Committee (IKDC) and Lysholm subjective questionnaires and were assessed using the Tegner activity scale preoperatively and at the final follow-up.

Clinician-applied varus stress radiographs at 20° knee flexion were obtained, and the SSD of the lateral joint opening was calculated as the closest perpendicular distance (in mm) between the lateral femoral condyle and the corresponding tibial plateau. This measurement was performed preoperatively and at least 1-year postoperatively.

**Fig. 2:**
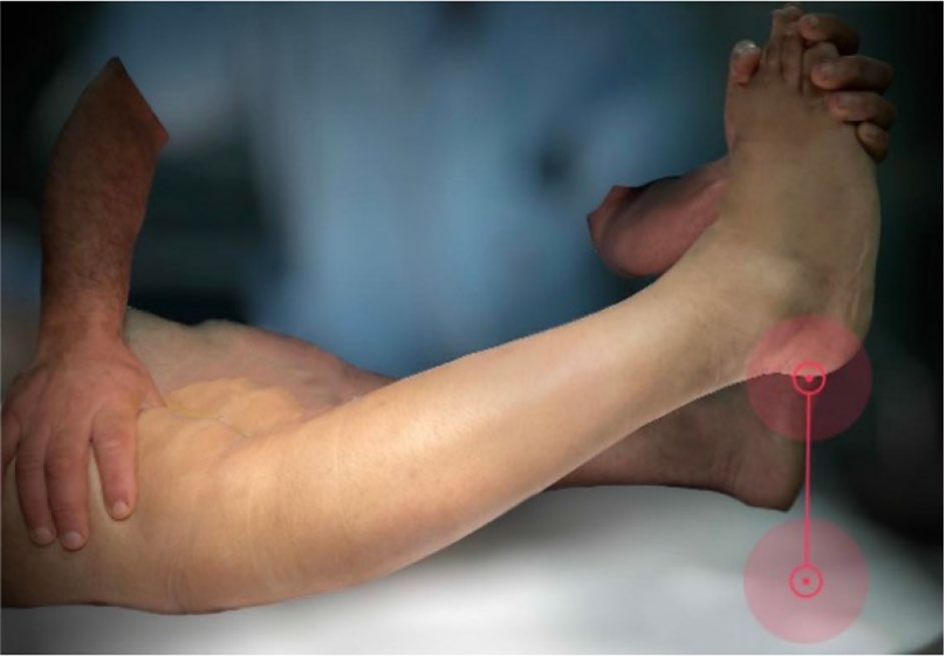
Measurement of the heel height as an indicator for the degree of knee hyperextension

The heel height was used as an indicator of the degree of genu recurvatum. The distance between the heel and examination table was measured (in cm) while the examiner lifted the big toe with one hand and firmly pushed the distal femur against the table with the other hand to prevent leg elevation and rotation (Fig. 2). The heel height SSD was measured preoperatively and postoperatively. External rotation (ER) stability was checked using a dial test performed at 30° of knee flexion in the prone position. The amount of external rotation was measured using a goniometer, and the external rotation angle SSD was calculated preoperatively and at the final follow-up. All pre-and post-operative clinical measurements were performed by a single orthopedic surgeon. The lateral joint opening on stress radiographs were measured independently by two orthopedic surgeons twice at an interval of six weeks, and the intra- and inter-observer agreements were assessed using interclass coefficient correlation.

### Modified anatomical PLC reconstruction with adjustable loop fixation to the tibia

After regional anesthesia was administered, the patient was positioned supine on the operating table. A padded pneumatic tourniquet was applied to the proximal thigh and inflated if necessary. A lateral post was mounted on the table at the level of the tourniquet together with a footrest, keeping the knee bent at 90° of flexion.

Arthroscopy was performed, and associated intraarticular injuries were addressed in a sequence (discussed later). The ipsilateral semitendinosus tendon was harvested using an open harvester in a standard manner. In cases of combined medial injury, the contralateral hamstring was harvested. The harvested tendon was whipstitched for 25 mm at either end with No. 2 Vicryl sutures (Ethicon, Somerville, NJ, USA) for shuttling into the tunnels.

A curvilinear skin incision was made over the lateral aspect of the knee, slightly anterior to the sagittal bisector, to facilitate femoral tunnel creation. The incision extended from the lateral femoral epicondyle to Gerdy’s tubercle on the anterolateral tibial surface. Full-thickness subcutaneous flaps were created posteriorly to expose the fibular neck. The common peroneal nerve was carefully dissected and released until it disappeared inside the peroneal muscle belly, anterior to the fibular neck.

The plane between the lateral head of the gastrocnemius and the LCL remnants above the biceps tendon was identified and bluntly dissected to allow access to the posterior tibia. A horizontal incision was made at the level of the fibular head through the biceps bursa; the LCL remnant was identified and tagged. A 6-mm fibular tunnel was drilled freehand at the fibular neck from an anterolateral starting point (champagne drop-off) toward a posteromedial exit point (downslope of the fibular styloid), slightly angulated in a distal to proximal direction. Careful sequential drilling was performed to avoid fibular head fractures. The passing suture was shuttled and clamped for subsequent use.

Thereafter, the tibial tunnel was drilled using a retro drill to the diameter of the doubled semitendinosus graft. The tunnel started at the soft spot, just medial to Gerdy’s tubercle on the anterolateral tibial surface and was aimed posteriorly to 1-cm medial to the proximal tibiofibular joint at the popliteus musculotendinous junction. A 25 mm socket was retro drilled, and a rigid loop was passed through the tunnel for graft shuttling. The iliotibial band was split horizontally at the level of the femoral attachment of the LCL and PT, and one socket (25-mm length and 6-mm diameter) was created at each anatomical footprint.

One free end of the graft was passed through the popliteal femoral tunnel and secured using a biocomposite screw (25 mm × 6 mm). The graft was then passed through the popliteal hiatus toward the posterolateral tibial side, exiting between the soleus and lateral heads of the gastrocnemius. The graft was subsequently passed through an adjustable loop and shuttled through the fibular tunnel. Thereafter, the adjustable loop was shuttled through the popliteal tibial tunnel, flipped over the anterior tibial cortex, and tensioned at 70° of knee flexion with neutral rotation. An interference screw (25 × 6 mm) was used to secure the PFL portion of the graft at the fibular tunnel at 70° flexion and neutral rotation, with maximal manual tension applied to the free graft end. The knee was cycled, and the adjustable loop was re-tensioned to tighten the PT-PFT.

The free end of the graft was passed deep to the superficial iliotibial band, superficial to the popliteus limb of the graft, and finally, shuttled through and fixed to the LCL femoral tunnel using a (25 mm × 6 mm) interference screw at 30° of knee flexion with slight valgus in neutral rotation. Anteroposterior and varus stability was confirmed and documented prior to wound closures. The wounds were copiously irrigated and closed in a standard fashion. A hinged knee brace was applied. The patients who underwent concomitant PCL reconstruction received an immobilization brace with a calf pad to support the tibia.

### Associated injuries

Meniscal injuries were addressed during arthroscopy before PLC reconstruction. Associated cartilage lesions were treated with microfracture or osteochondral autograft transfer when indicated. Associated PCL and ACL injuries were reconstructed in the same setting; PCL was reconstructed using an ipsilateral peroneus longus graft or contralateral hamstrings, and the ACL was reconstructed using an ipsilateral quadriceps graft. The cruciate ligaments were tensioned first, followed by the PLC.

### Postoperative rehabilitation

Weight-bearing was not allowed for the first 6 weeks. Thereafter, partial weight bearing was allowed aided by crutches for 2 weeks, followed by gradual progression to complete unaided weight bearing two months post-surgery. Range of motion (ROM) was restricted in a brace for the first 2 weeks, followed by a progressive increase in flexion motions up to 90° for 6 weeks postoperatively. Subsequently, unrestricted ROM was allowed, aiming for complete ROM recovery at 12 weeks post-surgery. Thereafter, a physiotherapist-supervised rehabilitation and conditioning program was initiated. Running without pivoting or cutting was allowed for approximately 6 months. Return-to-sports took 8–10 months after isokinetic testing and completion of a specific battery of tests by a physiotherapy team. Patients with concomitant PCL injuries were advised to wear a dynamic PCL brace starting from the 6th week for an additional 4 months.

### Statistical analysis

Data were analyzed using IBM SPSS (version 20.0; IBM Corp, Armonk, NY, USA). Means, standard deviations, and frequencies were calculated for the demographic data, stress radiograph results, ER angles, recurvatum angles, and subjective scores. The Shapiro–Wilk test was used to verify the normal distribution of the data. A paired Student’s *t* test was used to compare preoperative and postoperative Tegner scores, Lysholm subjective scores, and heel height measurements. The Wilcoxon test was used to compare preoperative and postoperative IKDC subjective scores, ER angle, and varus stress radiographs. The level of significance set at *p* < 0.05. Pearson’s and Spearman’s coefficients were used to correlate normally and nonnormally distributed quantitative variables. The SSD of the lateral joint line opening was measured independently by two observers, and the intraclass correlation coefficients indicated good inter- and intra-rater agreement for the radiographic measurements of varus laxity (0.82–0.76).

## Results

### Patient demographics

A total of 19 patients underwent surgeries between May 2018 and April 2020. One patient was lost to follow-up, and one patient experienced a traumatic event 11 months post-surgery and reported recurrent instability; thus, these patients were excluded from the study.

Seventeen patients who underwent modified anatomical PLC reconstruction using adjustable tibial fixation were available for final follow-up at an average of 25 months (range 24–28). The average patient age at the time of surgery was 29 years (range 19–42). All the patients were male, and the mean average time from injury to surgery was 14 weeks (range 3–40). Eleven patients were operated on within 6 weeks from the initial injury and classified as acute; six patients were operated on within 6 weeks to 12 months post-injury and were classified as chronic (Table 1).

**Table 1:** Patient demographics

Patient demographics (*n* = 17)	
Characteristic	Mean (range) or %
Age	29 (19–42) years
Follow-up period	25 (24–28) months
Time from injury to surgery	14 (3–40) weeks
Acute (< 6 weeks)	*N* = 11 (64.7%)
Chronic (≥ 6 weeks)	*N* = 6 (35.2%)
Sex	*N* = 17 males (100%)
Ligament injury pattern:	
PCL + PLC	N = 11 (64.7%)
PLC + ACL	N = 5 (29.4%)
Isolated PLC	N = 1 (5.8%)
Lower limb coronal alignment (HKA angle)	
Acute cases	− 3 (varus alignment)
Chronic cases	0 (Normal)

### Associated injuries (Table 2)

**Fig. 3:**
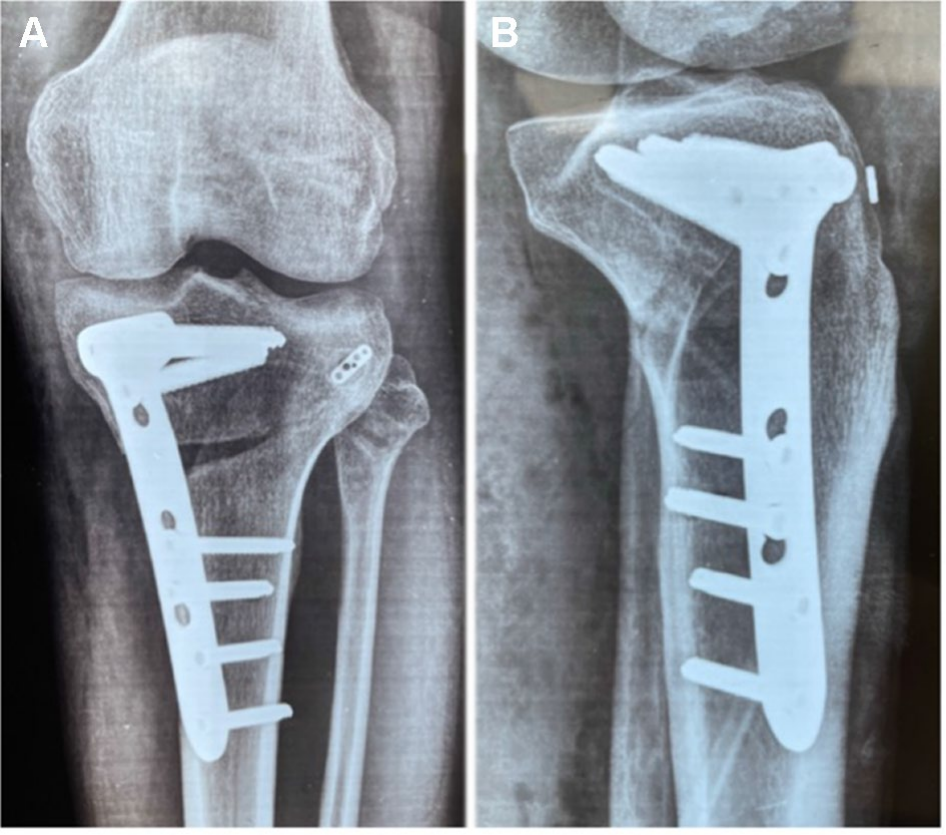
Postoperative X-ray showing concomitant high tibial osteotomy and posterolateral corner reconstruction in a double varus knee with varus thrust gait. **A** Anteroposterior view. **B** Lateral view showing the adjustable button sitting flush with the anterolateral aspect of the proximal tibia

Five patients underwent concomitant ACL reconstruction, and 11 underwent concomitant PCL reconstruction. Only one patient had an isolated chronic PLC injury at the time of presentation (the initial trauma-induced PCL injury had been conservatively managed and had healed adequately with stable posterior tibial translation measured on a stress radiograph) that underwent concomitant high tibial valgus-producing osteotomy due to varus thrust gait (Fig. 3). Eight patients had meniscal injuries and three had posterolateral capsular injuries. One patient had a distal avulsion of the biceps femoris tendon that was fixed using suture anchors. The iliotibial band was intact in all patients.

**Table 2:** Associated injuries and surgical procedures

Associated injuries	Surgical procedures	Number (%)
ACL tear	ACL + PLC reconstruction	*N* = 5 (29.4%)
PCL tear	PCL + PLC reconstruction	*N* = 11 (64.7%)
Double varus with varus thrust gait	High tibial osteotomy + PLC reconstruction	*N* = 1 (5.8%)
Medial meniscal tear		*N* = 6 (35.2%)
	Meniscal repair	*N* = 4 (23.5%)
	Partial meniscectomy	*N* = 2 (11.7%)
Lateral meniscal tear	Meniscal repair	*N* = 2 (11.7%)
Traumatic cartilage defect at the medial femoral condyle	Bone marrow stimulation	*N* = 4 (23.5%)
Biceps femoris tendon fibular avulsion	Refixation utilizing suture anchors	*N* = 1 (5.8%)
Posterolateral capsular injury	repair	*N* = 3 (17.6%)

### Patient-reported outcomes

At the final follow-up, all subjective clinical scores had improved postoperatively (Table 3). The mean IKDC and Lysholm scores significantly improved from 49.2 and 53 to 77.6 and 81.5, respectively. Although the mean preinjury Tegner activity scale score (6) decreased postoperatively at the final follow-up (5), it was not statistically significant.

**Table 3:** Comparison of pre- and postoperative PROMs and objective measures (mean ± SD)

Scores/objective measures	Preoperative		Postoperative at time of final follow-up		*p* value
	Mean ± SD	Range	Mean ± SD	Range	
IKDC	49.2 ± 11.2	33–68	77.6 ± 14.0	51–90	< 0.001*
Lysholm	53 ± 13.1	32–70	81.5 ± 8.4	65–96	< 0.001*
Tegner	6.6 ± 1.3 (preinjury)	4–8	5.8 ± 1.4	4–8	< 0.091
SSD of Lateral joint line opening	6.6 ± 2.1	3–10	3.4 ± 1.9	0–6	< 0.001*
SSD of ER angle	16.7 ± 3.8	12–23	3.5 ± 1.3	2–6	< 0.001*
Heel height SSD	6.8 ± 3.9	0–16	2.3 ± 1.3	0–4	< 0.001*

*Statistically significant

### Objective evaluation

**Fig. 4:**
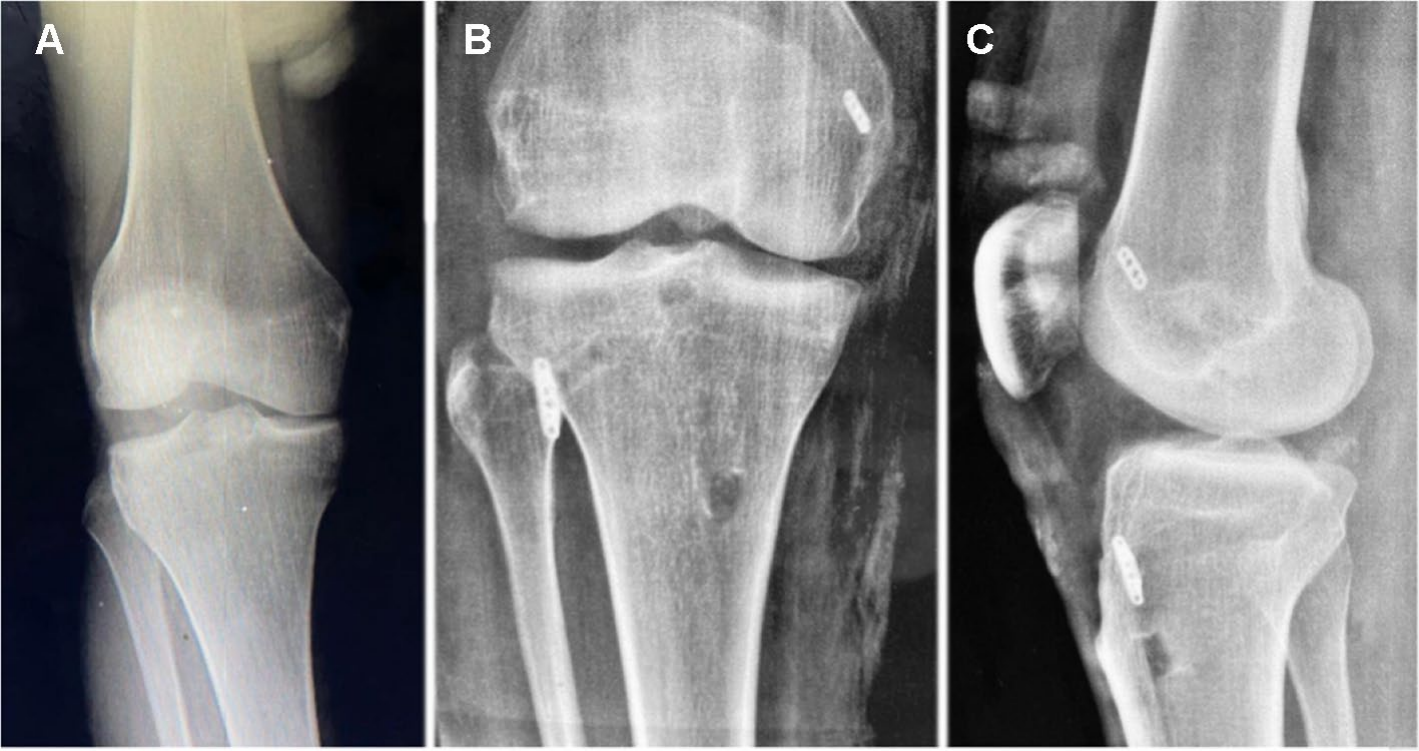
**A** Preoperative varus-stress radiograph of a patient with an acute combined PLC + PCL injury. **B** 1-yeay postoperative varus-stress radiograph with significant reduction of the lateral joint line opening to the normal contralateral value. **C** Lateral X-ray showing the position of the adjustable button at the anterolateral tibial surface. *PLC* posterolateral corner, *PCL* posterior cruciate ligament

**Fig. 5:**
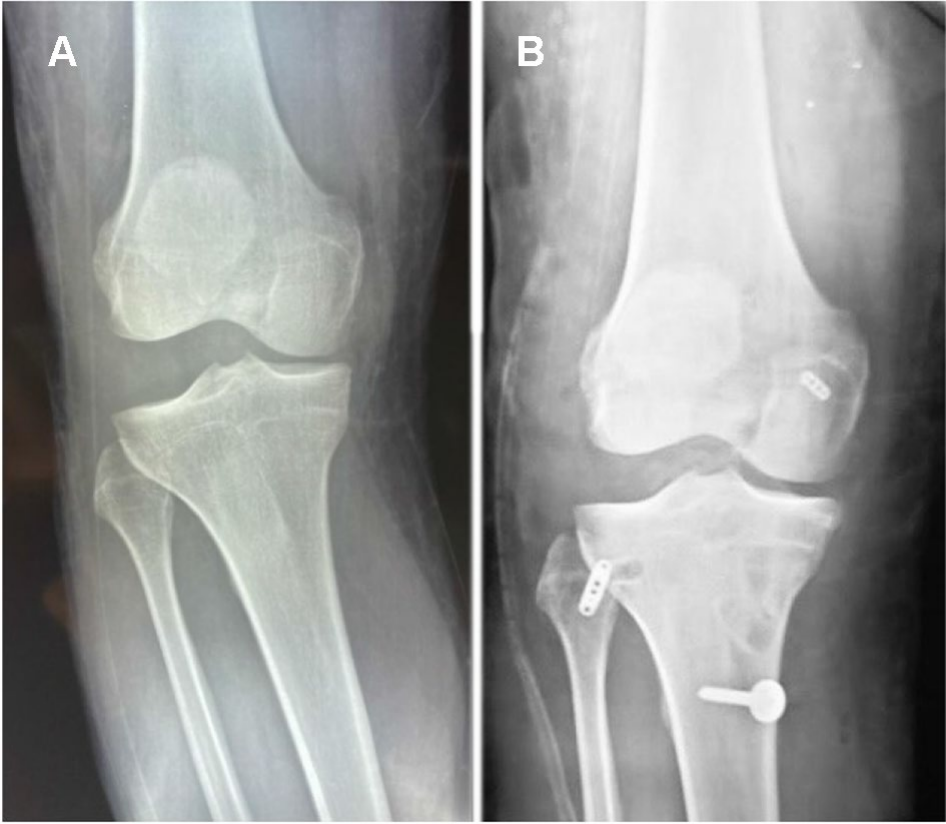
**A** Preoperative varus-stress radiograph of a patient with an acute combined PLC + PCL injury with high grade lateral laxity. **B** 1-year postoperative varus-stress radiograph. Although there was a substantial decrease in the lateral joint opening of > 10 mm, residual lateral laxity was detected clinically and radiologically. *PLC* posterolateral corner, *PCL* posterior cruciate ligament

All objective evaluation parameters had significantly improved postoperatively at the final follow-up (Table 3). There was a significant improvement in the mean lateral joint opening measured on the varus stress radiograph from 6.6 to 3.4; however, it remained more than the uninjured side (Figs. 4, 5).

Recurvatum was graded based on the SSD of the heel height as low grade (< 5 cm) or high grade (≥ 5 cm). Preoperatively, eight patients had low-grade recurvatum, and the remaining nine patients had high-grade recurvatum with SSD (≥ 5 cm). All postoperative genu recurvatum grades were low. The dial test was negative in all patients at the time of the final follow-up, with a marked improvement in the mean ER angle SSD from 16.7° to 3.5°. The average postoperative knee ROM was from − 4° to 134° of flexion. At the final follow-up, all the patients had a full range of motion equivalent to that of the contralateral healthy side.

There was no correlation between the final subjective outcomes (IKDC and Lysholm scores) and preoperative ER angle, recurvatum angle, and lateral joint line opening. Inferior subjective outcomes (IKDC and Lysholm scores) were associated with chronicity and combined PCL and PLC injuries (Table 4). The postoperative Tegner activity scale score correlated with the postoperative residual lateral laxity measured on varus stress radiographs. Hence, patients were unable to return to the pre-injury activity level in cases of residual significant varus laxity. However, postoperative varus laxity was positively correlated with the chronicity of the injury and preoperative varus laxity (Table 5), indicating that it was difficult to restore normal varus stability with the increase in time between injury and final surgery and in those with high preoperative varus laxity grades.

**Table 4:** PROMs according to ligament injury pattern and chronicity (mean ± SD)

Variable	ACL + PLC *N* = 5 (29.4%)	PCL + PLC *N* = 11 (64.7%)	*p* value
Postoperative IKDC	86.8 ± 3.5	73.2 ± 4.5	< 0.008*
Postoperative Lysholm	89.6 ± 0.5	78.1 ± 3.5	< 0.031*

Variable	Acute cases (< 6 weeks) N = 11 (64.7%)	Chronic cases (≥ 6 weeks) N = 6 (35.2%)	*p* value
Postoperative IKDC	86.8 ± 2.7	61.2 ± 16.2	< 0.01*
Postoperative Lysholm	87.6 ± 3.5	72.8 ± 9.8	< 0.01*

*Statistically significant

**Table 5:** Correlation between different variables

Spearman’s correlation	*r* value	*p* value
Pre-/postoperative lateral joint line opening measured on stress radiograph	0.818* CI 95% (0.54–0.93)	< 0.01*
Chronicity/residual lateral laxity	0.622* CI 95% (0.18–0.85)	< 0.02*
Postoperative Tegner scale/residual lateral laxity	− 0.883* CI 95% (− 0.96/−0.70)	< 0.001*

*Statistically significant

### Complications

Intraoperatively, there was a cut-through of the adjustable button during the final re-tensioning of the graft in one patient. The button migrated posteriorly, and the fixation was revised using an XL button. Two patients required manipulation under anesthesia to improve flexion range at 8 weeks post-surgery.

## Discussion

The most important finding of this study was that the modified anatomic PLC reconstruction technique with a single autograft significantly improved the subjective knee scores and objectively measured stability. However, varus stability was not completely restored to that in the non-operated knees.

Multiple techniques have been described for the reconstruction of the PLC. Laprade et al. [18] introduced the term “anatomical reconstruction,” where the three main static stabilizers (LCL, PFL, and PT) are reconstructed, thus theoretically restoring the native biomechanics. Most previous studies on anatomic PLC reconstruction used two separate allografts [6, 16, 18]. Due to allograft unavailability in some countries and the theoretical hazards of infection transmission, a reconstruction technique using two hamstring autografts has been described [19, 20]. Furthermore, because most PLC injuries are combined injuries that require other ligament reconstructions, techniques utilizing a single autograft have been described, such as using either a semitendinosus [21] or peroneus longus [22] graft.

In this study, we utilized an adjustable suspensory fixation on the tibial side, as described by Wood et al. [21] to artificially lengthen the autograft, independently tensioned the three different graft limbs, and re-tensioned the graft after knee cycling to eliminate creep in the construct.

Subjective improvement in patient-reported outcomes was statistically and clinically significant. The mean IKDC and Lysholm scores significantly improved from 49 and 53 to 77 and 81, respectively. These results are consistent with those of previous studies investigating the outcomes of anatomical PLC reconstruction. Laprade et al. [18] investigated a heterogeneous group of 54 patients who underwent anatomic PLC reconstruction with an average follow-up of 4.3 years; they determined that the average IKDC score at the final follow-up was 62. Franciozi et al. [23] reported the results of modified anatomical PLC reconstruction with mean postoperative IKDC and Lysholm scores of 70 and 81, respectively.

In this case series, inferior IKDC scores were associated with chronic and combined PCL injuries. Six patients had chronic injuries, with an average time of 46 weeks between injury and reconstruction. These cases reported average IKDC and Lysholm scores of 61 and 72, respectively, at the final follow-up. In a systematic review of the outcomes of chronically treated PLC injuries, Moulton et al. [24] reported that acutely treated injuries were associated with better outcomes than chronically treated injuries. Furthermore, chronic posterolateral instabilities treated with either auto- or allo-grafts, reportedly have mean postoperative Lysholm and IKDC scores of 65.5–91.8% and 62.6–86.0%, respectively, and a 10% failure rate based on objective stability. We believe that the longer the interval between injury and surgery, the longer the capsuloligamentous and tendinous structures on the lateral side of the knee remain stretched, which cannot be addressed by this type of reconstruction alone.

In this case series, 11 patients had an associated PCL injury, with average postoperative IKDC and Lysholm scores of 73 and 78, respectively. These results were worse than those for ACL-based injuries. These results are similar to those reported by Feucht et al. [25] who showed that ACL-associated injuries led to superior patient-reported outcomes and earlier return to work than PCL-associated injuries. Franciozi et al. [23] showed inferior outcomes in PCL-associated PLC injuries, with mean postoperative IKDC and Lysholm scores of 63 and 78, respectively, compared to ACL-associated injuries (82 and 87). These inferior outcomes may be due to the excessive force applied on the reconstructed PLC and PCL grafts, as they both act against posterior tibial translation [5, 26]. Furthermore, the posterolateral joint capsule is also usually injured; therefore, there are no intact structures to resist the posterior translation early in the postoperative phase, which may lead to graft stretching.

In our study, although SSD of lateral joint opening measured on stress varus radiographs improved significantly from an average of 6.6 mm preoperatively to 3.4 mm postoperatively, varus stability was not completely restored. Residual lateral laxity could be detected clinically and radiologically. This residual varus instability was strongly correlated with the amount of preoperative lateral joint line opening measured on stress radiographs, the interval between injury and surgery, and the postoperative Tegner activity scale.

A number of clinical studies reported residual varus laxity after anatomical PLC reconstruction [15, 23, 27]. Van Gennip et al. [15] noticed an improvement in varus stability using the Larson fibular sling reconstruction technique compared to the Laprade anatomical reconstruction technique; however, other studies showed no such difference [12, 28]. Possible reasons for this residual varus laxity may be the use of a single graft limb to reconstruct the LCL or the weakness of the part used to reconstruct the LCL, which tapers and thins at both ends.

In contrast to previously published data [23], we found a strong correlation between preoperative and postoperative lateral joint line openings measured on stress radiographs. Thus, this reconstruction technique could not completely restore varus stability in those with high-grade varus laxity. Therefore, for patients with a large preoperative lateral joint line opening (> 10 mm), we recommend augmenting this type of reconstruction by repairing the injured ligaments, utilizing a strip from the biceps tendon, adding a synthetic internal brace, or using a two-tailed fibular sling to reconstruct the LCL in addition to PT reconstruction.

Although there was a significant improvement in the Tegner activity scale score from the preoperative to the postoperative state, most patients did not return to their pre-injury sports participation level. This finding is in line with that of Van der Wal et al. [27] who recommended counseling patients that they may not return to their pre-injury athletic levels. In addition, we found that the degree of residual varus laxity measured on stress radiographs was a strong predictor of postoperative activity.

All patients in this study showed significant improvement in the recurvatum and external rotation angles postoperatively, returning to values close to the contralateral uninjured side. Although some clinical studies have underestimated the value of reconstructing the PT [12, 28], based on previous biomechanical studies [17, 25, 29], we believe that PT and PFL reconstruction is crucial in controlling tibial external rotation. This is because the PT acts statically and dynamically to limit hyperextension and posterior tibial translation, especially in PCL injuries.

Hamstring autografts are successfully utilized to reconstruct the PLC [20, 23]. The use of a single semitendinosus graft may be beneficial, particularly for multiligament injuries or when allografts are unavailable. An adjustable loop was used to lengthen the graft artificially, as described by Wood et al. [21], in this study. We found it beneficial to independently tension the components of the construct and re-tension them after knee cycling. The main disadvantage of this technique is the need for a sufficiently long graft (> 25 cm), which may be unavailable. In such cases, the use of two separate grafts for anatomical PLC reconstruction is recommended.

Our study has several limitations. This study had a limited sample size with heterogeneous surgical procedures used to treat associated injuries, which are typical for such injuries which presenting as part of combined knee injuries. This was a case series study with no control or comparison groups. We used historical controls for other techniques, with an inherent bias in comparisons owing to different sampling, inclusion criteria, and associated injuries. In addition, no gold standard for a specific stress radiographic technique or magnitude of varus force application during testing has been established to assess knee stability. Thus, there may be a bias in measuring the lateral joint line opening using stress radiographs. To minimize this bias, stress radiographs were obtained by the same clinician, and preoperative and postoperative clinical evaluations were performed by the senior author. The effect of the posterior tibial slope on clinical outcomes was not studied in this cohort, which may have contributed to the residual laxity detected in the PCL reconstruction group.

## Conclusions

PLC reconstruction with a hamstring autograft using a modified anatomical reconstruction technique significantly improved the subjective patient scores and the objective knee stability. However, varus stability was not completely restored compared to the uninjured knees.

## Data Availability

Due to the sensitive nature of the data, information created during and/or analysed during the current study is available from the corresponding author on reasonable request to researchers who provide a methodologically sound proposal.

## Abbreviations

ACL: Anterior cruciate ligament
PCL: Posterior cruciate ligament
PLC: Posterolateral corner
LCL: Lateral collateral ligament
PFL: Popliteofibular ligament
PT: Popliteus tendon
SSD: Side-to-side difference
ER: External rotation
ROM: Range of motion
